# Understanding Auto-Brewery Syndrome in 2023: A Clinical and Comprehensive Review of a Rare Medical Condition

**DOI:** 10.7759/cureus.37678

**Published:** 2023-04-17

**Authors:** Jananthan Paramsothy, Sai Dheeraj Gutlapalli, Vijay Durga Pradeep Ganipineni, Ikpechukwu J Okorie, Derek Ugwendum, GianPaolo Piccione, James Ducey, Gnama Kouyate, Arnold Onana, Louis Emmer, Vaithilingam Arulthasan, Philip Otterbeck, Jay Nfonoyim

**Affiliations:** 1 Internal Medicine, Richmond University Medical Center Affiliated with Mount Sinai Health System and Icahn School of Medicine at Mount Sinai, Staten Island, USA; 2 Internal Medicine Clinical Research, California Institute of Behavioral Neurosciences and Psychology, Fairfield, USA; 3 General Medicine, SRM Medical College Hospital and Research Center, Chennai, IND; 4 General Medicine, Andhra Medical College/King George Hospital, Visakhapatnam, IND; 5 Internal Medicine, Thomas Hospital Infirmary Health, Fairhope, USA; 6 Endocrinology, Richmond University Medical Center Affiliated with Mount Sinai Health System and Icahn School of Medicine at Mount Sinai, Staten Island, USA; 7 Pulmonary and Critical Care, Richmond University Medical Center Affiliated with Mount Sinai Health System and Icahn School of Medicine at Mount Sinai, Staten Island, USA

**Keywords:** gut microbiomes, candida infections, alcohol use disorder (aud), auto-brewery syndrome, metabolism of alcohol

## Abstract

Auto-brewery syndrome (ABS) occurs when the gastrointestinal tract produces excessive endogenous ethanol. This article examines various aspects of ABS such as its epidemiology, underlying etiology, diagnostic difficulties, management strategies, and social implications. By synthesizing the existing medical literature, we hope to identify understanding gaps, pave the way for further research, and ultimately improve detection, treatment, and awareness. The databases we used are PubMed, PubMed Central, and Google Scholar. We carefully screened all published articles from inception till date and narrowed down 24 relevant articles. We at Richmond University Medical Center and Mount Sinai are one of the leading medical centers for diagnosing and treating this rare condition in the United States.

## Introduction and background

In auto-brewery syndrome (ABS), endogenous ethanol production from the carbohydrates ingested that is fermented by yeast, mainly *Candida* species, within the gut [1]. This condition is also described in the literature as gut fermentation syndrome [2,3]. Patients usually present with dizziness, disorientation, and impaired motor skills [4]. In most cases, ABS is diagnosed by ruling out other causes and treated with diet changes and antifungal medications [5].

Although relatively unknown, ABS has historical roots dating back several decades [6]. It was reported for the first time in 1948 that humans produce endogenous alcohol in their gastrointestinal tract [6]. The first reported case describes the fatal gastric rupture in a five-year-old boy caused by autonomous alcohol production [6]. According to some evidence, the sweet potato dinner produced gas under such pressure that the stomach ruptured, releasing alcohol into the peritoneum [6].

Several studies in the Japanese literature have documented instances of ABS [7]. In Japanese, it is known as Meitei-sho [7]. One of the first cases of ABS was documented in 1972, involving two patients who showed symptoms of intoxication despite not drinking [7].

Over the years, there has been a gradual increase in awareness of this condition [5]. There was a notable study done in 2000 about a child with short-bowel syndrome (short-gut syndrome) who also had ABS [5].

Methods

For our literature review, we conducted the data search by using the MeSH strategy and used the keywords “Auto-Brewery,” “Auto-Brewery Syndrome,” “Diagnosing Auto-Brewery Syndrome,” “Treatments for Auto-Brewery Syndrome,” and “Alcohol Fermentation Syndrome.” The databases used were PubMed, PubMed Central, and Google Scholar. We screened through all the studies from inception till date and gathered 24 articles relevant to our literature review.

## Review

Epidemiology and prevalence

**Figure 1:**
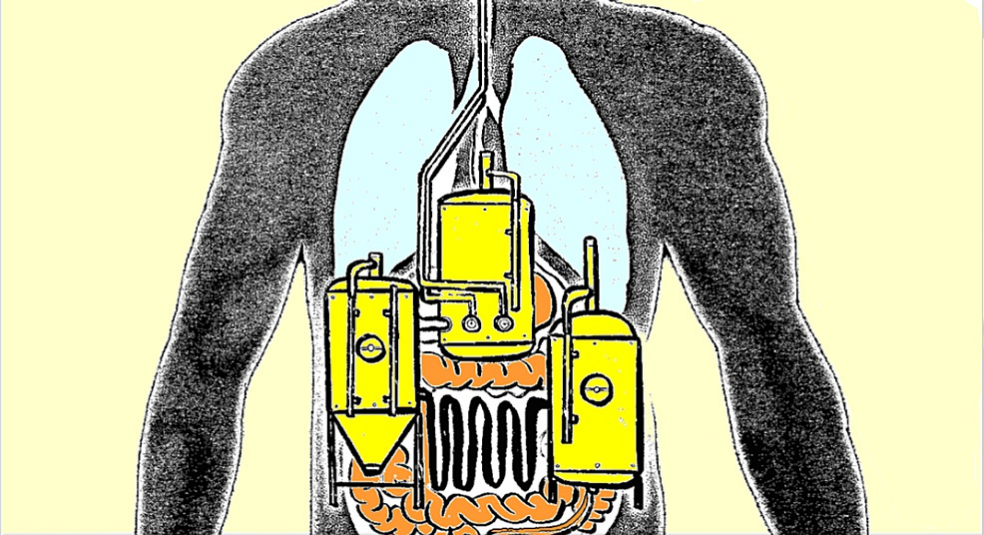
Auto-brewery syndrome Image Credits: Vijaya Durga Pradeep Ganipineni and Sai Dheeraj Gutlapalli

Although epidemiological data on ABS are limited, small-scale studies and case reports suggest that its prevalence is significantly underestimated [8]. Because of the absence of standardized diagnostic criteria and nonspecific presentation of the syndrome, ABS can be very difficult to clinically diagnose and may be misdiagnosed [9]. It is crucial to determine its true prevalence and improve patient outcomes [9]. Some studies have reported an association between patients with diabetes and cirrhosis. Hafez et al. examined ABS in diabetic and liver patients, finding higher blood ethanol concentrations than in healthy controls [10]. Hafez et al. compared the blood ethanol level of 50 patients with liver cirrhosis and diabetes to healthy subjects after 12 hours of fasting. All participants never consumed alcohol prior to the study. Blood ethanol levels were significantly higher in patients with liver cirrhosis and diabetes than in those in the control group. Blood ethanol concentrations were different between the groups which suggests there might be a different metabolic pathway involved; in addition, there is no correlation noticed between blood glucose levels and blood ethanol levels suggesting that endogenous ethanol production is not affected by meals in these groups. In their study, ABS is more prevalent in diabetic and cirrhotic patients, emphasizing its importance as a cause of the pseudo-toxicity of ethanol and implications for diagnosis and treatment [10]. Figure 1 shows a graphical representation of the ABS.

**Figure 2:**
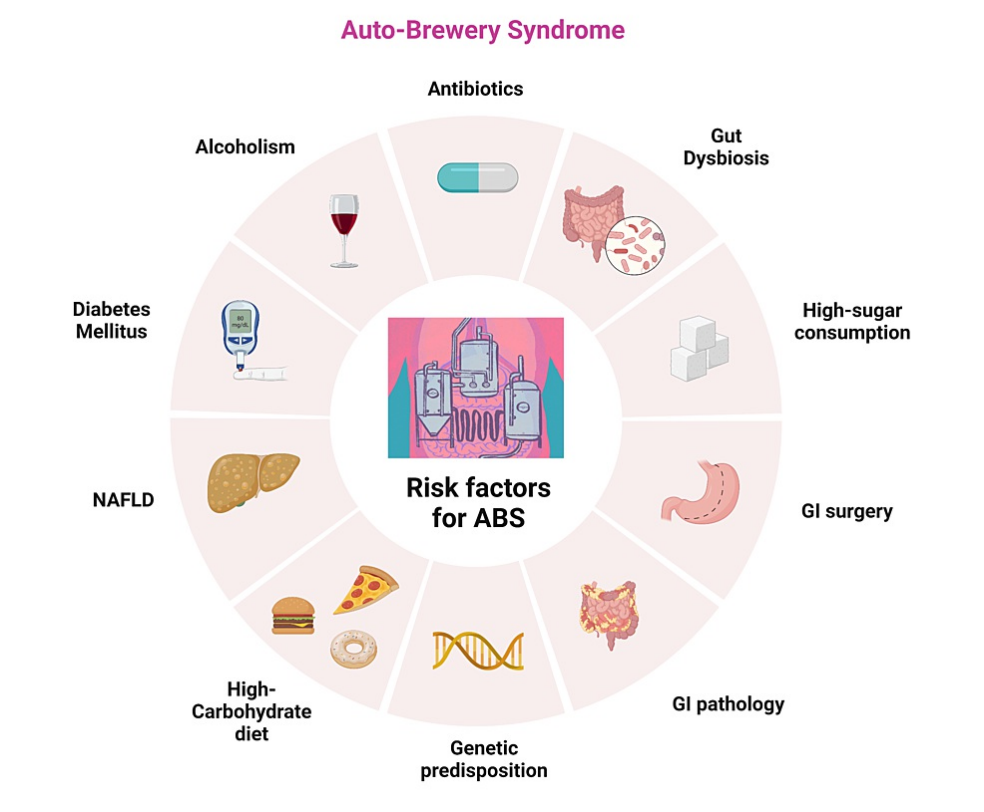
Risk factors for auto-brewery syndrome Image Credits: Vijaya Durga Pradeep Ganipineni and Sai Dheeraj Gutlapalli

A study conducted by Simic et al. examined whether endogenous ethanol production has medicolegal implications in patients with diabetes. Despite not drinking alcohol, individuals with diabetes mellitus can produce detectable amounts of ethanol. Therefore, traditional alcohol testing methods can be challenged, complicating forensic investigations and legal proceedings [11]. Welch et al. presented a case study of a 71-year-old man with Crohn's disease and ABS. In their study low-carbohydrate diet and antibiotic avoidance reduced ABS recurrence [12]. The risk factors associated with ABS are listed in Figure 2.

Causes and pathophysiology of ABS

ABS is caused by the overgrowth of fermenting microorganisms, primarily *Saccharomyces cerevisia*e, in the gastrointestinal tract [10]. Endogenous alcohol production occurs when these microorganisms break down carbohydrates into ethanol, leading to intoxication without drinking alcohol [10]. The risk factors for ABS include gastrointestinal abnormalities, prolonged antibiotic use, and diets that promote microbial fermentation [12-14].

High-Carbohydrate Diets

Carbohydrate-rich diets increase the production of ethanol in the gastrointestinal tract because carbohydrates provide additional substrates for fermentation by gut microbes. Cordell and McCarthy reported that a low carbohydrate diet improved symptoms in a patient with ABS, implying that carbohydrate intake plays a role in ABS [4].

Role of the Gut Microbiome in ABS

The gut microbiota consists of a wide variety of microorganisms that are important for maintaining health. In particular the gut microbiome plays a crucial role in the development of both innate and adaptive immunity, and that the immune system also helps to shapes the microbiome.

Microbiome research has advanced over the years, and it has helped us better understand gut microbes and host health. Kashyap et al. used genetically modified mice to investigate to find how host specific gene mutation alters the gut carbohydrate concentration that could influence gut microbiome composition and function, in addition how changes in diet influence this effect. The gene mutation which this study involved is the Fut-2 gene which encodes an enzyme called galactoside 2-α-L-fucosyl-transferase-2, which is responsible for the addition of terminal fucose residues to certain carbohydrates. Alterations in this gene significantly change the gut carbohydrate landscape. Their study found that the specific Fut-2 gene mutation of the host and changes in diet to high carbohydrate content worked hand in hand to influence the composition of the intestinal microbiota. These findings imply that customized dietary interventions may be required to alter the gut microbiota and alleviate symptoms of ABS [13].

According to Eaton and Howard, gut fungal dysbiosis may be the underlying cause of gastrointestinal symptoms in some patients. This imbalance in the intestinal microbiota is associated with gastrointestinal and systemic diseases. The authors found that many patients experienced relief through dietary intervention alone, while a minority required antifungal therapy. However, the reintroduction of certain foods into the diet led to symptom recurrence in more than 50% of the patients, with specific trigger foods varying among individuals. Understanding the mechanisms of dysbiosis and its role in disease development is crucial for developing effective treatments for diseases related to gut dysbiosis [14].

Genetic Predisposition to ABS

Alcohol use disorders have led to extensive research on alcohol dehydrogenase (ADH) and aldehyde dehydrogenase (ALDH) enzymes in the body. In healthy individuals, ADH is the primary enzyme involved in the breakdown of ethanol in acetaldehyde and ALDH, which responsible for breaking down acetaldehyde to acetate which is a harmless substance which can be further metabolized and eliminated by the body. These enzymes are important for understanding the ABS and finding the genetic linkage associated with the disease. Research shows that individuals with genetic polymorphisms of ADH and ALDH can find it more difficult to metabolize ethanol, which can worsen alcohol intoxication symptoms [15]. Therefore, the interaction between the ADH and ALDH genes and their polymorphisms can contribute to the severity and development of ABS.

Antibiotic usage and the risk of ABS

In several case reports, prior antibiotic use has been linked to ABS. In a 2006 case report, a patient with chronic intestinal pseudo-obstruction exhibited elevated levels of blood ethanol without alcohol consumption. The overgrowth of *Candida albicans* and *S. cerevisia*e within the gastrointestinal tract was attributed to the administration of amoxicillin and clavulanic acid along with a sugar-rich diet [16].

Malik et al. presented further evidence of the potential link between antibiotic use and ABS. In their case report after three weeks of three times a day 250 mg of Cephalexin administration, the patient in this study developed ABS symptoms. The case report suggested antibiotics disrupt the intestinal microbiota, which allows yeast species to proliferate and ferment carbohydrates into ethanol, resulting in ABS [1].

Welch et al. study shows alteration in the gut dysbiosis with the prolonged antibiotic usage. Their study found that long term use of as amoxicillin clavulanic acid, and metronidazole can cause bacterial overgrowth in the small intestine [12]. In addition to gut dysbiosis, persons who consume higher amounts of carbohydrates gives favorable environment yeasts, such as *S. cerevisiae*, to grow, which in turn ferment the carbohydrates and produces alcohol [12].

In the case described by Saverimuttu et al., the patient's history of antibiotic treatment with amoxicillin/clavulanic acid after nasal septal and dental surgery may have contributed to the development of ABS. Accurate diagnosis and treatment of ABS are essential to significantly improve the quality of life of patients with ABS. Taking into account the link between antibiotic use and ABS, further research is needed to understand the complex interactions between antibiotics, gut dysbiosis , and the development of ABS [8].

Microbiome involved in ABS

**Figure 3:**
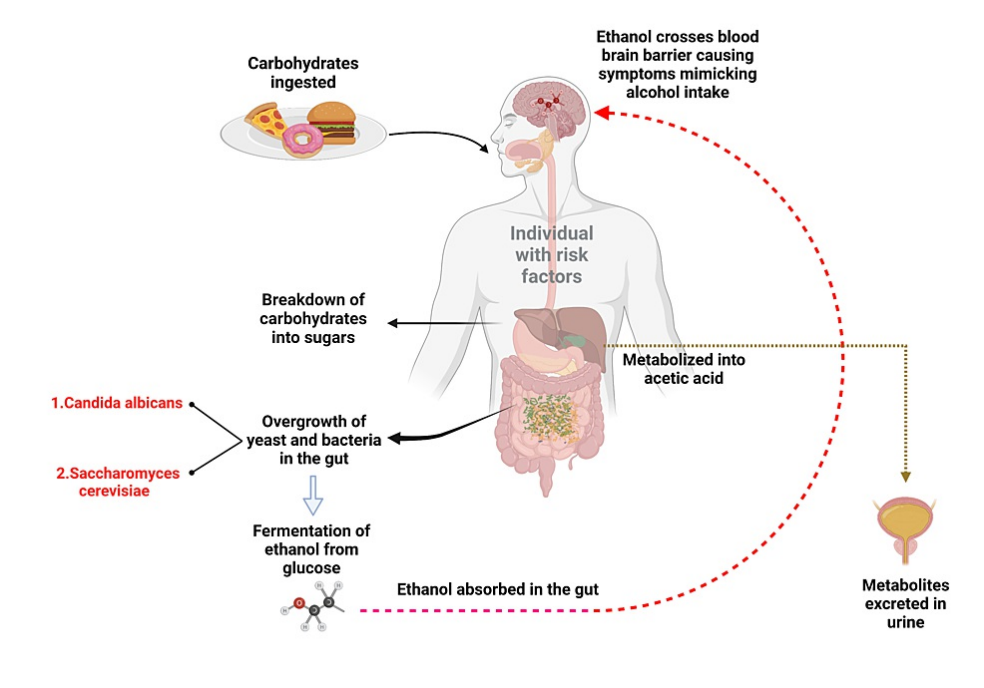
Various patho-physiological mechanisms of auto-brewery syndrome Image Credits: Vijaya Durga Pradeep Ganipineni and Sai Dheeraj Gutlapalli

Bayoumy et al. identified specific microorganisms responsible for ABS based on a systematic review of case reports of ABS. The review found that ABS is primarily caused by yeast recolonization. The ABS cases were associated with *S. cerevisiae*, *Saccharomyces ​​​​​​boulardii*, *C. glabrata*, *C. albicans*, *C. kefyr*, and *C. parapsilosis* [17]. *C. albicans*, which is more prevalent in immunosuppressed individuals, can also cause ABS [5]. ABS case has also been linked to *S. boulardii*, a probiotic yeast [16]. Pathophysiological mechanisms of ABS are elaborated in Figure 3.

Diagnosis

Due to its rarity and overlapping symptoms with other conditions, ABS requires a detailed diagnosis. A multifaceted diagnostic approach helps to ensure that ABS is accurately identified and distinguished from other conditions with similar presentations, allowing for appropriate treatment and management. A detailed patient history, including dietary habits, alcohol consumption, and gastrointestinal symptoms, is crucial. A comprehensive physical examination is essential to detect unexplained ethanol intoxication. Some patients experience symptoms more frequently if they suffer from intestinal obstruction, gastroparesis, or liver dysfunction in addition to ABS. ABS can be difficult to diagnose because its symptoms are similar to those of alcohol abuse or psychiatric disorders. An accurate measurement of blood alcohol concentration and a controlled carbohydrate diet are necessary for the diagnosis of ABS [1].

Symptoms and signs

ABS usually presents with unexplained intoxication, dizziness, disorientation, and ataxia without alcohol consumption [17]. In addition to chronic fatigue, some patients with ABS may experience bloating and irritable bowel syndrome (IBS) [1,5].

The symptoms of ABS include memory loss, mental changes, recurrent seizures, slurred speech, and comatose states. Some people report feeling faint, nauseated, vomiting, having difficulty articulating, or experiencing blurred vision [7]. In addition to incoherent speech and glassy eyes, there may also be frequent falls [8]. To try to improve the basic quality of life of those affected by ABS, it is crucial to be aware of their symptoms and to obtain an early diagnosis and effective treatment. The various organ systems and the corresponding symptomatology is elaborated in Table 1.

**Table 1:** System-wide symptoms of auto-brewery syndrome

Systems	Symptoms
Nervous System	Memory loss, Mental status changes, Recurrent seizures, Slurred speech, Incoherent speech, Difficulty in articulation, Blurred vision, Dizziness, Disorientation, Ataxia
Gastrointestinal	Bloating, Belching, Nausea, Vomiting.
Musculoskeletal	Poor coordination, frequent falls, Stumbling gait.
General	Unexplained intoxication, Glassy eyes, Alcohol smell in breath, Chronic fatigue.

Laboratory tests and diagnosis of ABS: a comprehensive approach

Diagnostic approach includes complete blood counts (CBC) and comprehensive metabolic panels (CMP). Basic diagnostic tests to rule out other differential diagnoses that can present with the similar symptoms and signs. In addition, stool testing can assist in the screening process, although small amounts of fungal colonization in the lower gastrointestinal tract can be considered normal [18].

Monitoring blood alcohol levels can be useful in identifying ABS. If blood alcohol levels are elevated in the absence of alcohol consumption, ABS may be present [1]. To corroborate a patient's denial of alcohol consumption, it is essential to consult a family member or friend [19]. Confirming a diagnosis of ABS requires excluding other conditions. Additional tests or consultation with specialists may be necessary to eliminate differential diagnoses.

Controlled carbohydrate challenge

The carbohydrate challenge test has proven to be effective in identifying individuals with ABS [9]. Carbohydrate challenge tests involve administering a specific carbohydrate load (commonly glucose) to patients and monitoring their blood alcohol levels over a period of time. It has been shown to significantly raise blood alcohol levels in people with ABS, as carbohydrates trigger endogenous ethanol production [1,8,12]. Table 2 describes the steps of using carbohydrate challenge test in diagnosing ABS.

**Table 2:** Steps of using carbohydrate challenge test in diagnosing auto-brewery syndrome The table adapted from the information from Malik et al. (2019) [1] and Saverimuttu et al. (2019) [8].

Step	Procedure	Details
1	Patient Preparation	48-hour alcohol abstinence, 8 hour fasting
2	Baseline Measurements	Obtain patient demographic and clinical information. Measure initial blood alcohol levels and test for alcohol metabolites (ethyl glucuronide and ethyl sulfate) in urine samples.
3	Carbohydrate Load Administration:	Administer an oral carbohydrate load containing 100-200 grams of glucose
4	Blood Alcohol Level Monitoring	Monitor blood alcohol levels at predetermined intervals (30 min, 1 hr, 2 hr, 4 hr, 8 hr, 12 hr, 24 hr) Continue monitoring for a total of 24 hours or until the test is halted due to elevated blood alcohol levels
5	Data Analysis	Assess the blood alcohol level data for significant elevation in the absence of exogenous alcohol consumption.

Differential diagnosis

The diagnosis of ABS often mimics other gastrointestinal and liver conditions. IBS, for example, can cause bloating and diarrhea. Small intestinal bacterial overgrowth (SIBO) can cause abdominal pain and bloating, making it an important condition to consider during diagnosis [20]. In patients with ABS, it is possible that patients can present with extensive liver dysfunction and hepatic encephalopathy.

Legal and social implications

The impact of ABS on driving under the influence (DUI), employment, and personal relationships is significant. Several DUI cases have reported that ABS defendants were acquitted of DUI charges due to the impact of the condition on blood alcohol content (BAC) readings [21].

The legal system struggled to distinguish between alcohol use disorders and endogenous ethanol production caused by ABS [21]. Individuals with ABS may face unjustified accusations of alcoholism, which can lead to employment problems. Additionally, the unpredictable nature of ABS can lead to poor job performance and negatively impact employment prospects.

Furthermore, stigmatization can lead to job loss of work and diminished career prospects [21]. The stigma associated with intoxication-like symptoms and frequent misdiagnosis of ABS as alcohol abuse can also affect personal relationships [9]. Relationships may be strained due to mistrust and misunderstanding between family members and friends. ABS has far-reaching consequences for affected individuals, affecting their legal status, employment opportunities, and personal relationships. It is possible to mitigate these adverse effects by increasing awareness and understanding of ABS.

Treatment and management

Lifestyle Modifications

Recent research on ABS highlights the need for lifestyle modifications to manage this rare condition. Low-carbohydrate and sugar-restricted diets are essential for reducing fermentable substrates that promote endogenous ethanol production [1,9]. The literature varies among case-to-case basis in treating ABS with a low-carbohydrate diet; however, a diet that emphasizes high quality meats and low starchy vegetables appears to be effective in managing the symptoms [12,22]. The diet should be based on individuality and focused on patient other comorbidities. 

Therapeutic Interventions

ABS is a rare medical condition in which the body produces alcohol internally due to sugar fermentation by the overgrowth of yeast or bacteria in the gastrointestinal tract [22]. This article explores various treatment strategies for ABS, including pharmacological interventions, dietary modifications, and probiotic and fecal transplant therapies.

Pharmacological Interventions for ABS: Pharmaceutical treatments for ABS primarily target underlying fungal overgrowth with antifungal medications. There are few side effects associated with nystatin, which has been proven to be safe [22]. Fluconazole and itraconazole can be prescribed if nystatin therapy fails or is inappropriate. Micafungin can be administered intravenously if the oral treatment fails [22,23].

In most of the cases that was reported in the literature ABS was treated with oral fluconazole 100-150 mg a day for 14 days alone [4,5,16,23], and oral fluconazole is usually the first line of treatment in these cases. Nystatin 50,000 IU three times for 10 days alone or with fluconazole 150 mg was given if there in some treatment resistant cases with fluconazole [1,22]. Micafungin 150 mg IV can be given if the oral treatment fails [1,8]. Table 3 gives a brief summary of the treatment options for ABS.

**Table 3:** Summary of treatments and dosages for auto-brewery syndrome

Treatment	Dosage
Oral fluconazole (100-150 mg/day)	14 days
Nystatin (500,000 IU, 3x/day)	10 days
Intravenous micafungin (150 mg/day)	6 weeks
Probiotic (L. acidophilus)	6 weeks
Multi-strain probiotic	1.5 years

Dietary modifications for ABS: To reduce alcohol production in the gastrointestinal and genitourinary tracts, ABS patients with ABS should adhere to a high protein, low carbohydrate diet [22]. It is possible to tolerate carbohydrates found in fruits and vegetables in small amounts, but nutritionists should be involved in the management of ABS to help patients avoid carbohydrates as much as possible [22]. For the initial treatment of ABS, it is recommended that you refrain from eating carbohydrates for six weeks, since fungi convert carbohydrates into alcohol [23].

Probiotic and fecal microbiota transplant in ABS: Probiotic therapy, such as Lactobacillus acidophilus, can be used along with antifungal treatment in ABS [1,23]. Long-term probiotic therapy will be beneficial after completion of antifungal treatment [1,23].

Fecal microbiota transplantation (FMT) has been suggested as a potential treatment for ABS to restore the balance of intestinal bacteria. According to a study published in the Annals of Internal Medicine, a 47-year-old man treated with ABS treated with FMT remained symptoms free for 36 months after treatment [24]. More research is needed to evaluate the safety and efficacy of FMT before it becomes a standard treatment option [24].

## Conclusions

ABS is a rare and potentially underdiagnosed condition characterized by endogenous ethanol production in the gastrointestinal tract, resulting in symptoms of intoxication without alcohol consumption. There are potential risk factors involved in causing ABS. Medical conditions such as diabetes mellitus, liver cirrhosis, and Crohn's disease are associated with ABS. Diets rich in carbohydrates, gut dysbiosis, and prolonged antibiotic therapy, genetic polymorphisms in ADH, ALDH enzymes can contribute to developing ABS. Even though several microbial species are described in the literature, *S. cerevisiae* and *C. albicans* are the common yeast species implicated in ABS. Diagnosing ABS can be a clinical challenge, due to its nonspecific presentation, and overlap with other gastrointestinal and liver disorders. For an accurate diagnosis, a comprehensive approach must be taken, including detailed patient history, physical examination, laboratory tests such as blood cell counts, metabolic panels, stool cultures, and controlled carbohydrate challenge tests. Early identification and intervention will be necessary to significantly improve the quality of life of patients with ABS.

A multifaceted approach to the treatment is essential, including dietary modifications, pharmacological interventions, and probiotics, to effectively manage ABS. A low-carbohydrate, sugar-restricted diet reduces the fermentable substrates in the gut that promote endogenous ethanol production. Many antifungal and antibacterial medications can effectively reduce yeast overgrowth and ethanol production, especially antifungals like fluconazole, Nystatin, and Micafungin. It is possible to reduce intestinal yeast colonization by using probiotics, especially Lactobacillus and Bifidobacterium. In addition to ABS's impact on patient health, it also has implications on patient lifestyle including impaired job performance, and social stigmatization, ABS has significant implications for DUI cases and personal relationships. It is vital to raise awareness and knowledge of ABS among medical professionals, legal entities, and the public to mitigate these adverse consequences and ensure fair treatment for those affected. Further research is required to determine the true prevalence of ABS, develop standardized diagnostic criteria, and investigate optimal treatment strategies. Ongoing advancement in microbiome research will help us to understand the complex interactions between antibiotics, gut microbes, and ABS development.
